# Percutaneous vertebroplasty (PVP) to treat specialized type of endplate fractures around the Schmorl’s node: a prospective study of 65 patients

**DOI:** 10.1186/s13018-020-01873-6

**Published:** 2020-09-10

**Authors:** Yaoshen Zhang, Peng Yin, Jincai Yang, Yong Hai

**Affiliations:** Department of Orthopedics, Beijing Chao-yang Hospital, Capital Medical University, Beijing, 100020 China

**Keywords:** Schmorl’s nodes, Endplate fractures, Percutaneous vertebroplasty (PVP), Minimally invasive surgery, Low back pain

## Abstract

**Background:**

The Schmorl’s nodes (SNs) are defined as the herniation of the intervertebral disc into the vertebral body. Endplate fractures around the Schmorl’s node could result in severe back pain that is similar to vertebral compression fractures. The objective of this study was to prospectively analyze the surgical effectiveness and safety of percutaneous vertebroplasty (PVP) for endplate fractures around the SNs.

**Methods:**

Seventy-one consecutive patients with the fresh endplate fracture around SNs from October 2017 to February 2018 were enrolled in this study. The visual analog scale (VAS) and Oswestry disability index (ODI) scores of low back pain were evaluated in all patients preoperatively, postoperatively, and at 1 month, 6 months, and 1 year after primary single level PVP. Surgery-related data including duration of the operation, injected cement volumes, and surgical complications were recorded.

**Results:**

Sixty-five patients with fresh endplate fractures around the SNs were treated successfully via percutaneous vertebroplasty. Our study showed that the VAS scores and ODI scores of patients were obviously improved after operation. Cement leakage into the disc space occurred in 5 patients (7.7%), and adjacent segment refractures occurred in 2 patients (3.1%). No other surgical complications, including infections or nerve root injuries were encountered.

**Conclusions:**

Based on the results of this prospective study, PVP was an effective and safe procedure for endplate fractures around the SNs.

**Trial registration:**

ChiCTR, ChiCTR1800016453. Registered 2 June 2018—retrospectively registered,

http://www.chictr.org.cn/com/25/historyversionpuben.aspx?regno=ChiCTR1800017602

## Background

Schmorl’s nodes (SNs), defined as herniation of the discs into the vertebral body through the endplate, were firstly described by the Christian Georg Schmorl in 1927 [1]. SNs are common spinal lesions in the asymptomatic population. The prevalence of SNs varies from 2 to 76% [2–4] with increased frequency in male population (76% of cases) [5]. SNs are more frequent in the thoracolumbar region (T7-L1) and are more likely to locate on the inferior surface of the vertebral body (62.3%) [6].

The pathophysiological mechanism(s) of SN formation remains controversial. Recent studies show that SN development is related to subchondral osteonecrosis and abnormal development of the vertebra blood vessels [7, 8].

The majority of SNs are asymptomatic in the healthy population and do not require treatment. However, specific types of SNs are associated with refractory low back pain, which is ineffective for conservative treatment. A few studies have reported that Schmorl’s nodes (SNs) may be related to degenerative disc diseases and osteoporotic vertebral compression fractures, and lead to low back pain [9–13]. Patients receiving spine MRI show low signal intensity on T1WI, and high signal intensity on T2WI in the endplate around the SNs are at increased risk to appear as low back pain. These changes in spinal MRI indicate an acute stage edema in the bone marrow surrounding the SNs [12].

According to previous studies, SNs may represent the early stages of endplate osteoporotic fractures [14]. Epidemiological studies have shown that patients with SNs have a higher risk of appear symptomatic vertebral compression fractures in the endplate around the SNs compared to the general population [14, 15]. Long-term follow-up studies suggest that approximately 26% of SNs progress in size and 13% show significantly increased T2 signals in MRI. Such subgroups of SNs are more likely to occur vertebral compression fractures [13]. Endplates surrounding the SNs with high signal intensity on fat-suppression sequences in spinal MRI indicate an acute stage of bone marrow edema, resulting in severe back pain that is similar to acute vertebral compression fractures. Vertebral compression fractures are common in the elderly population [16, 17], and PVP could obtain satisfactory clinical results. To date, the outcomes of painful SNs with endplate fractures treated by PVP are less well studied and limited to retrospective studies of small sample sizes [18–24]. We therefore prospectively analyzed 65 cases of endplate fractures around the SNs who underwent PVP in our institution. The objective of the study was to prospectively analyze the surgical effectiveness and safety of PVP for SNs with endplate fractures.

## Materials and methods

### Patient population

Seventy-one consecutive patients with the fresh endplate fracture around SNs from October 2017 to February 2018 were enrolled in this study. All patients were treated at the Department of Orthopedics of the Affiliated Chaoyang Hospital of Capital Medical University.

### Inclusion and exclusion criteria

**Fig. 1:**
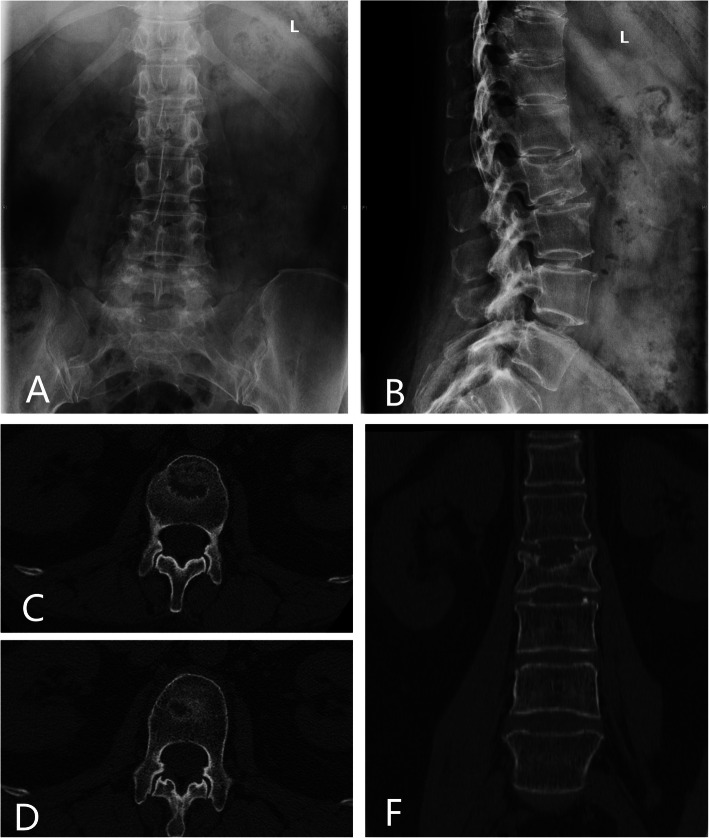
Anteroposterior (**a**) plain X-ray of lumbar spine showed no abnormalities. Lateral plain X-ray (**b**), axial (**c**, **d**), and coronal (**f**) CT images of the lumbar spine demonstrate a Schmorl’s node in the inferior endplate of L2

**Fig. 2:**
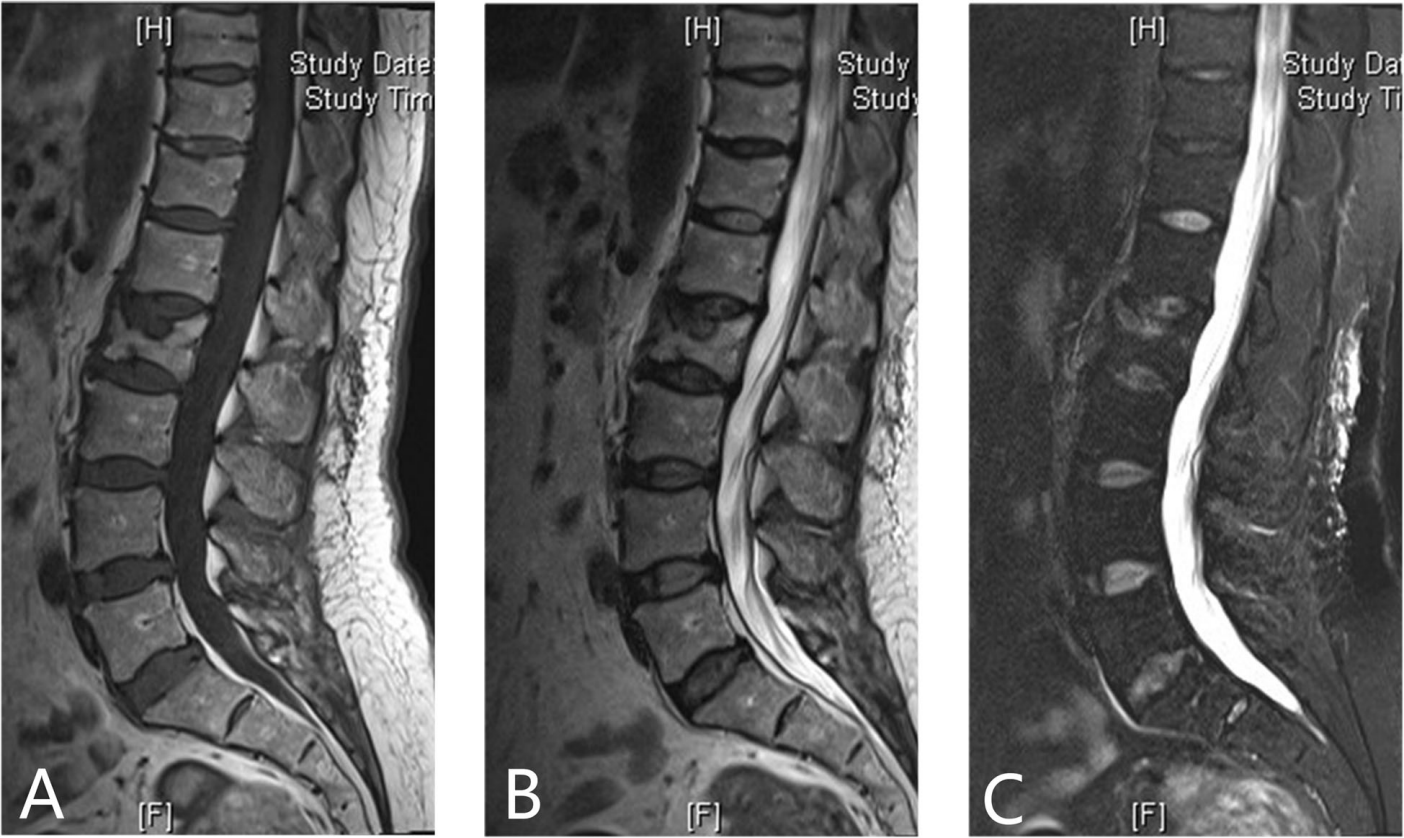
Pre-operation sagittal lumbar MRI demonstrates a large SN at the inferior endplate of L2 on T1WI and T2WI sequences (**a**, **b**), and high signal intensity in T2 inferior endplate around SN on fat-suppression sequences (**c**)

**Fig. 3:**
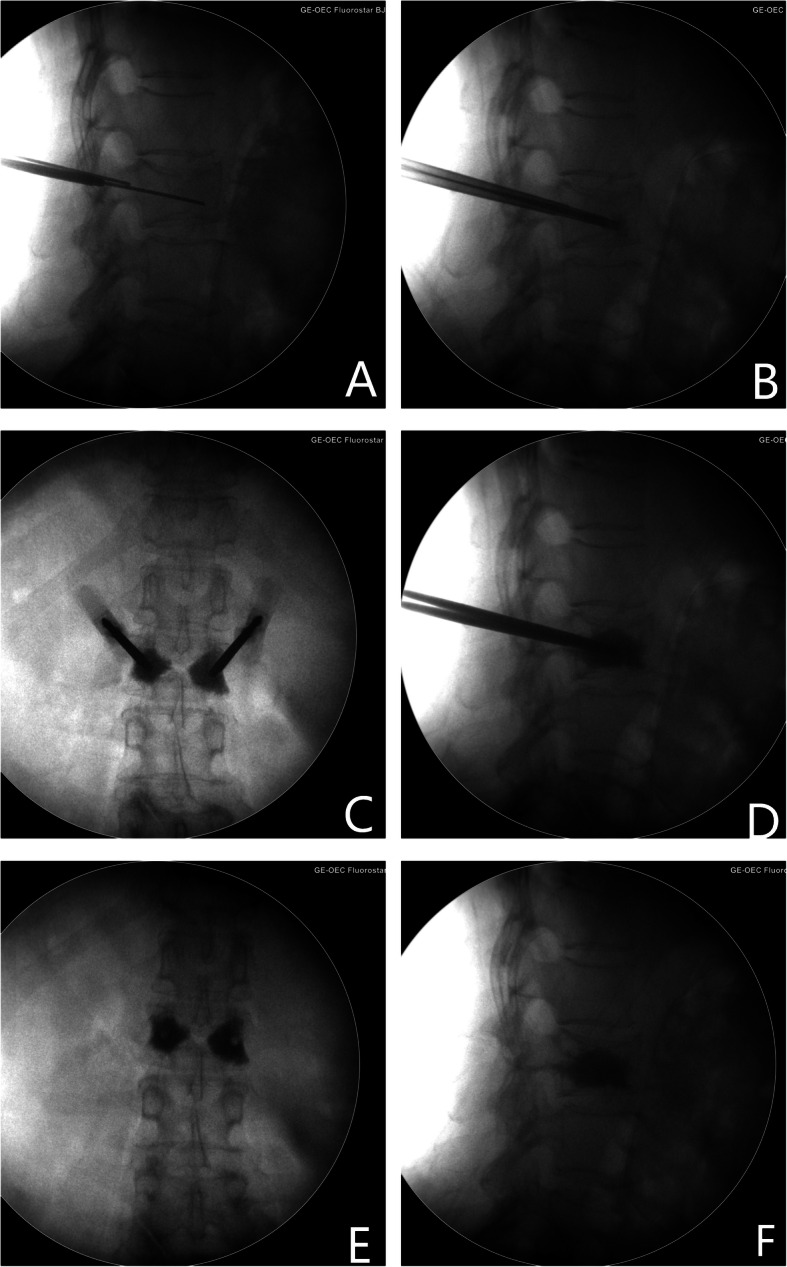
Intra-operation spinal anterior-posterior and lateral plane X-rays. First, bilateral needles were inserted through the pedicle towards the endplate around the SN (**a**, **b**). Then, high-viscosity polymethylmethacrylate (PMMA) was carefully injected towards the profile of the SNs under fluorescence (**c**, **d**). Post-operation anteroposterior and sagittal X-ray of lumbar spine demonstrate PMMA filled the inferior endplate of L2 vertebral body around SN (**e**, **f**)

The inclusion criteria of our study were (1) all these patients received conservative treatments such as bed resting, medication, and physical therapy for 2 weeks after they were diagnosed as VCFs. Only for these patients who were resistant to conservative treatments, surgical interventions were then performed; (2) SNs at the thoracic or lumbar spinal vertebral body confirmed by preoperative computed tomogram (CT)(Fig. 1) and MRI (Fig. 2); (3) spine magnetic resonance imaging showing high signal intensity on fat-suppression sequences in the single-level endplate around SNs with or without low signal intensity on T1WI and high signal intensity on T2WI; (4) the treatment of percutaneous vertebroplasty alone; (5) regular follow-ups after discharge.

The exclusion criteria: (1) patients with leg pain; (2) previous lumbar surgery history in the level with SN; (3) multi-level endplate fractures; (4) CT and MRI revealing patients with other causes of low back pain including spinal disc herniation, lumbar spinal stenosis, degenerative disc diseases, and spinal malignant lesions.

The study was approved by the local Ethics Committee of Beijing Chaoyang Hospital, Capital Medical University, and written consent was obtained from all the patients.

### Operative techniques

All patients received bilateral PVP procedures by one senior spine surgeon. Initially, patients were placed in the prone position on a Jackson table, and spinal anterior-posterior and lateral plane X-rays were imaged using a C-arm angiographic unit to determine the entry point. After disinfection, the surgical area was draped in a sterile manner. Local anesthesia was performed with 1% lidocaine, bilateral needles were inserted through the pedicle, and carefully moved towards the endplate surrounding the SN according to pre-operative X-ray, CT, and MRI (Fig. 3 a, b). Next, the spinal needle was removed, and a small skin incision was introduced at the entry point. Two tapered tubes were then inserted along the guidewire targeted at impaired endplate around the SN. Bone marrow and tissue biopsies around the SN were taken with biopsy forceps. Finally, high-viscosity polymethylmethacrylate (PMMA) was carefully injected towards the profile of the SNs under fluorescence (Fig. 3 c, d). Working tubes were then removed and the two incisions were sutured (Fig. 3 e, f).

During the post-operation period, all patients were told to wear the lumbar or thoracic-lumbar brace for at least 1 month after the surgery and take anti-osteoporotic medicine for as long as possible.

### Clinical evaluation

Clinical data of the included patients were collected. The visual analog scale (VAS) for back pain, Oswestry disability index (ODI), and Modified MacNab criteria of the patients were collected prior to surgery and during follow-up (1 m, 6 m, 12 m). Clinical questionnaires were completed and collected through the outpatient service or by telephone.

### Radiographic evaluation

**Fig. 4:**
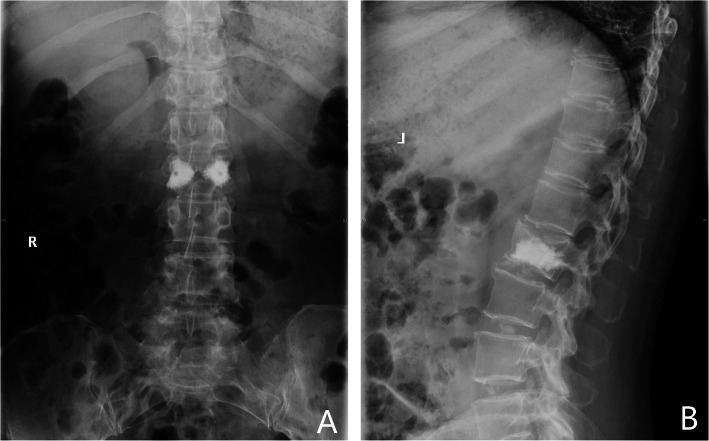
Anteroposterior and sagittal X-ray of lumbar spine demonstrate PMMA filled the L2 inferior endplate and vertebral body around SN at 1 month

**Fig. 5:**
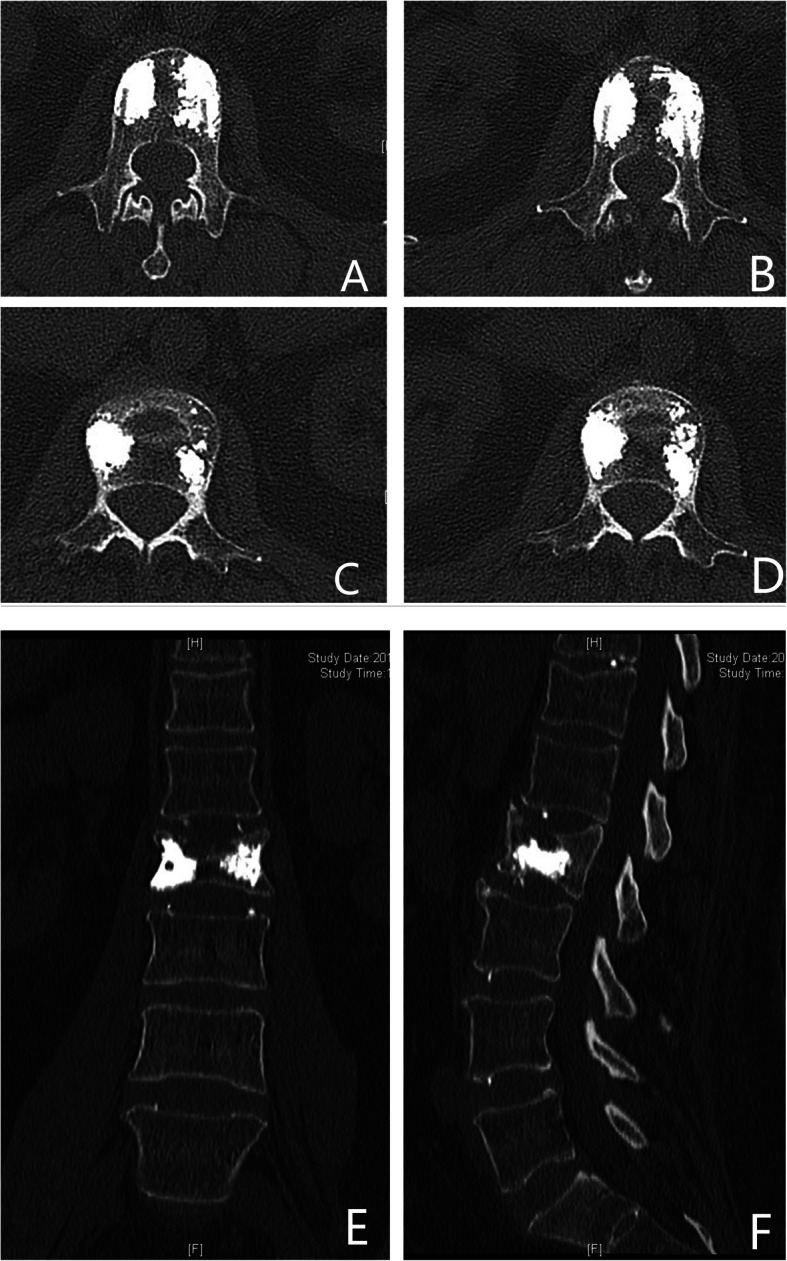
Axial CT radiography showed polymethylmethacrylate (PMMA) filled around the profile of the SNs in an eggshell shape (**a**–**d**). Anteroposterior and sagittal CT showed PMMA filled the profile of the SNs in L2 around SN without cement leakage at 1 month

Spinal radiographs (X-ray and CT) of all patients were evaluated prior to surgery and during follow-up (1 m, 6 m, and 12 m) (Figs. 4 and 5).

### Data analysis

Patient data was analyzed using SPSS software (version 22.0, SPSS Inc., Chicago, IL, USA). The mean ± standard deviation (SD) was used to express continuous variables. To analyze categorical variables, chi-square analyses and Fisher’s exact tests were employed. A paired *t* test was used for comparisons of the VAS and ODI scores at each time-point pre- and post-operation. A *P* value less than 0.05 was considered statistically significant.

## Results

### Demographics and surgical data

Seventy-one patients who underwent single-level PVP for endplate fractures around the SNs and who met the inclusion criteria were initially included. Six patients were excluded as they failed to complete regular follow-up. Twenty-seven male (41.6%) patients and 38 female (58.4%) patients were finally enrolled. The mean age at the time of surgery was 72.93 years old (range 49 to 90 years old) with an average follow-up period of 14.82 months (range 12 to 16 months). For 65 patients who included in this study, 53 patients get a pre-operation bone mineral density test, among them, 47 was diagnosed as osteoporosis, 3 was diagnosed as osteopenia, and rest 2 was diagnosed as normal.

At the surgical level, there was 1 case (1.5%) at T7, 4 cases (6.2%) at T8, 5 cases (7.7%) at T9, 9 cases (13.8%) at T10, 11 cases (16.9%) at T11, 9 cases (13.8%) at T12, 8 cases (12.3%) at L1, 7 cases (10.8%) at L2, 6 cases (9.2%) at L3, 2 cases (3.1%) at L4, and 3 cases (4.6%) at L5. Among these patients, 38 (58.5%) nodes were located at the inferior endplate while 27 (42.5%) were located at the superior endplate. The mean surgical duration was 34.29 min (ranging from 21 to 53 min), and the average injected cement volume was 4.88 ml (ranging from 1.5 to 6 ml). Cement leakage into the disc space occurred in 5 patients (7.7%), and adjacent segment refractures were found in 2 patients (3.1%). No other surgical complications including infections or nerve root injuries were found (Table 1).

**Table 1:** Clinical features of the patients (±SD)

Clinical features	
Variation	Value
No. of cases	65
**Baseline date**	
Male	*N* = 27(41.6%)
Female	*N* = 38(58.4%)
Age	72.93 ± 10.251
Follow-up (month)	14.82 ± 2.556
BMI	26.59 ± 5.527
**Surgical level**	
T7	*N* = 1(1.5%)
T8	*N* = 4(6.2%)
T9	*N* = 5(7.7%)
T10	*N* = 9(13.8%)
T11	*N* = 11(16.9%)
T12	*N* = 9(13.8%)
L1	*N* = 8(12.3%)
L2	*N* = 7(10.8%)
L3	*N* = 6(9.2%)
L4	*N* = 2(3.1%)
L5	*N* = 3(4.6%)
**Location of SN**	
Inferior endplate	*N* = 38(58.5%)
Superior endplate	*N* = 27(42.5%)
**Surgical procedure**	
Duration of operation (min)	34.29 ± 10.839
Cement volume (ml)	4.88 ± 0.91
**Macnab score**	
4(excellent)	*N* = 32(49.2%)
3(good)	*N* = 25(38.5%)
2(fair)	*N* = 8(12.3%)
1(poor)	*N* = 0(0%)
**Complications**	
Adjacent vertebral fractures	*N* = 2(3.1%)
Cement leakage	*N* = 5(7.7%)

### Clinical evaluation

The mean VAS scores were documented prior to operation: 4 h, 1 month, 6 months, and 12 months post-operation. Following PVP surgery, the VAS scores improved from the pre-operative average of 7.80 to 2.08 at 4 h, 1.42 at 1 month, 1.18 at 6 months, and 1.23 at 12 months post-operation. The ODI scores improved from the preoperative average of 68.69 to 27.03 at 1 month post-operation (Table 2).

**Table 2:** Comparison of the VAS and ODI scores of the patients between pre- and post-operation ($\overline{\mathrm{x}}$ ± SD)

	**VAS score**	***P*** **value**
Pre-operation	7.80 ± 1.438	
Post-operation (4 h)	2.08 ± 0.797	< 0.001
Post-operation (1 m)	1.42 ± 0.496	< 0.001
Post-operation (6 m)	1.18 ± 0.391	< 0.001
Post-operation (12 m)	1.23 ±0 .430	< 0.001
	**ODI score**	***P*** **value**
Pre-operation	68.69 ± 1.288	
Post-operation (1 m)	27.03 ± 0.763	< 0.001

According to the modified MacNab criteria, the outcomes were recorded and were classified 1 month post-operation, as 4 points in 49.2% of the patients (excellent, 32 cases), 3 points in 38.5% of the patients (good, 25 cases), 2 points in 12.3% of the patients (fair, 8 cases), and 0 point in 0% of the patients (poor, 0 cases).

## Discussion

SNs are common spinal lesions in the general population, in which the majority are asymptomatic and require no treatment [2–4]. However, patients with endplate fractures around the SNs could result in acute back which is refractory to conservative treatment. Our results showed that PVP was an effective and minimally invasive surgery for this special type of endplate fracture.

Chronic low back pain commonly originates from the intervertebral disc, facet joint, sacroiliac joint, and other spinal anatomic structures. Any spinal structures that are innervated by nerves could become source of pain if tissue damage occurs. According to previous studies, SNs could cause severe back pain through the release of inflammatory mediators or cause adjacent endplate fractures [7, 12]. Although the precise relationship between SNs and back pain remain unclear, a specific subgroup of patients with SNs whose spinal MRI showed a high signal intensity on fat-suppression sequences in the vertebral endplate surrounding the SNs, appear to have severe low back pain that originated from the edematous vertebral endplate [18–24]. The same signal changes in MRI images were defined as inclusion criteria in our study.

An array of studies attempted to elaborate the possible etiology of SN development. To date, no consensus has been reached. Recent studies show that SNs development may be related to subchondral osteonecrosis of the endplate followed by disc materials protruding into the weakened vertebral endplate [7]. Others suggest that SNs correspond to an abnormal development of the vertebra blood vessels. The abnormal presence of vertebral vessels in the adult population may weaken the vertebral endplate, resulting in development of SNs [8]. Observational imaging study have demonstrated a possible relationship between vertebral fractures and SNs. Patients with a history of vertebral fractures during childhood were at increased risk of developing SNs at the index fracture level [15]. In our study, 41 patients did not have a clear history of trauma. Because for patients with severe osteoporosis, they do not need a clear traumatic history to develop VCFs, and slight impacts to the spine such as coughing and lifting heavy stuff could lead to bone fractures and result in severe back pain. Based on previous studies, patients with painful SNs who are refractory to conservative treatment show improvement through several surgical procedures including discoblock, percutaneous vertebroplasty, spinal fusion, and tumor necrosis factor alpha (TNF-a) inhibition [18–26]. The use of these surgical options is based on the causative factors of low back pain, generated from endplate fractures or from degenerative disc materials that result in a special type of discogenic back pain.

SNs could cause chronic back pain through inflammatory mediators and cytokines, which are produced by degenerative disc (annulus rupture) and osteonecrosis of the endplate [26]. Pathophysiological mechanisms of painful SNs are identified with the degenerative disc diseases, resulting in a special type of discogenic back pain, in which provocative discography is typically positive and spinal MRI shows a low signal intensity on T1WI, and a high signal intensity on T2WI (Mordic-I change), indicating degenerative changes of the disc material [25]. Surgical procedures including spinal fusion and discoblock can be used to manage this type of painful SN [25, 26].

Another mechanism of painful SNs are associated with occult trabecular bone fractures in the endplate around the SNs. In the general population, the vertebral body and endplate are composed of trabecular bones. However, for some patients with SNs, the normal trabecular bony structure is impaired due to subchondral osteonecrosis or traumatic history [7, 8, 15]. It is important to note the characteristic changes in spine MRI. Impaired endplates around the SNs usually result in high signal intensity on fat-suppression sequences of spine MRI, indicating an acute stage of bone marrow edema, similar to the pathophysiological changes observed for vertebral compression fractures. Instability, inflammatory mediators, and mechanical pressure over the edematous endplate can induce acute and severe back pain [12]. A long-term improvement of vertebroplasty in patients with osteoporotic fractures has been shown [27–29]. PVP could relieve the back pain generated from the impaired endplates by stabilizing fractures around the SNs. Besides, high temperature generated from the period of PMMA solidificated could kill nerves around the impaired endplate, which may also contribute to pain relief.

To date, a paucity of studies have reported the outcome of this specific subgroup of painful SNs with endplate fractures treated by vertebroplasty [18–24].

Painful SNs treated by PVP were first described in 2006 by Masala and colleagues in 18 patients with SNs who showed an improvement in their back pain after PVP [22]. Subsequently, Markus Wenger and coworkers reported a case of painful SNs treated by PVP under computer navigation to access the node without entering the cavity of the SNs [20]. Recently, Sun Zhi-Yong and colleagues reported 32 patients who were successfully treated with percutaneous balloon kyphoplasty (PKP). The height of the vertebral body restoration and functional improvements were maintained during a mean follow-up period of 5 years [19]. Some studies suggest to insert the working cannulas directed towards the apex of the SNs to fill the polymethylmethacrylate (PMMA) around the profile of the SNs in an eggshell shape [18, 21]. However, studies highlighting these procedures are limited to retrospective studies study [18–24]. Hence, in our study, we treated 65 patients with severe back pain by PVP that were suspected to be secondary to fresh endplate fractures (with high signal intensity on fat-suppression sequences in spinal MRI) around SNs. Clinical data from these patients was prospectively collected. The VAS and ODI scores significantly improved after PVP and were well maintained in the mean post-operative follow-up of 14.82 months. Cement leak into the disc space occurred in 5 patients (7.7%) without symptomatic leakage. Adjacent segment refractures occurred in 2 patients (3.1%), and all were managed successfully after secondary PVP procedures. No other surgical complications were found.

Endplate fractures around the SNs differ from those of common osteoporotic vertebral compression fractures. In our experience, surgical technique tips and specific radiographic change should be paid more attention to. Firstly, fresh endplate fractures must be confirmed by preoperative magnetic resonance imaging (MRI) with high signal intensity on fat-suppression sequences around SNs. For the surgical technique, cement should be injected towards the high signal intensity on fat-suppression sequences area in the endplate around the SN to stabilize and fill the occult trabecular bone fracture. This tough eggshell-shaped cement around the SNs can prevent progression of SNs and minimize refractures of the endplate. In the period of PMMA injection, surgeon should perform carefully and gently with frequent X-ray imaging examinate to evaluate the diffusion of cement and prevent cement leakage.

To our knowledge, this is the first prospective study that has reported the outcome of the use of PVP for the treatment of fresh endplate fractures around the SNs. However, small series cases were included in our study; hence, a prospective study with large sample should be performed to investigate our results.

## Conclusions

Endplate fractures around the SNs are common in daily clinical practice, and their surgical strategy differs from common osteoporotic vertebral compression fractures. Thus, the characteristic changes of the SNs in spine CT and MRI should be noticed. Based on this prospective study for this special type of endplate fracture, PVP represents an effective and safe procedure.

## Data Availability

The datasets used and/or analyzed during the current study are available from the corresponding author on reasonable request.

## Abbreviations

SNs: Schmorl’s nodes
MRI: Magnetic resonance imaging
PKP: Percutaneously kyphoplasty
PMMA: Polymethylmethacrylate
VAS: Visual analog scale
ODI: Oswestry disability index
